# Self-Reported Sleep during the COVID Lockdown in a Sample of UK University Students and Staff

**DOI:** 10.3390/healthcare10102083

**Published:** 2022-10-19

**Authors:** John H. Foster, Sandra Rankin

**Affiliations:** 1 Alcohol Policy and Mental Health Studies, School of Health Sciences, University of Greenwich, London SE10 9LS, UK; 2 School of Human Sciences, University of Greenwich, Old Royal Naval College, 150 Dreadnought, Park Row, London SE10 9LS, UK

**Keywords:** Nottingham Health Profile, alcohol, gender, sleep dysfunction, health, well-being

## Abstract

The link between disturbed sleep and the extended lockdown period resulting from COVID-19 is well established. Data from an online survey of 2341 of university students (n = 1972, 84.2%) and staff were reported. Overall (n = 1710, 73.1%) were female and the mean age for the sample was 29.26 (SD = 12.86). 1799 (76.8%) provided self-reported data from the Nottingham Health Profile (NHP) Sleep Subscale that allowed sleep to be compared prior to the lockdown period and during the lockdown period. Sociodemographic data which included, gender, age, whether an individual was a student or member of the university staff, ethnicity, caring responsibilities, and highest educational level were collected. Other data included, the NHP Sleep Sub-scale, change in alcohol consumption during the lockdown period, routine behaviours during the lockdown period, self-efficacy and health and wellbeing. There was a significant deterioration in NHP Sleep scores (*p* < 0.001) and all areas of sleep that were assessed significantly deteriorated during the lockdown period. These included indicators of sleep quality, sleep latency, sleep duration, sleep disturbance and increased use of sleep medication. Following a multinomial logit regression with change of NHP sleep scores entered as the dependent variable there were several significant predictors. Women had greater sleep dysfunction than men. Increased alcohol consumption, lower educational status and a deterioration in health and well-being scores were associated with greater sleep dysfunction. Not having a designated area to work in and not putting on clothes and make-up were both associated with greater sleep dysfunction during the lockdown period. These findings confirm the importance of taking steps to maintain sleep hygiene during extended lockdown periods.

## 1. Introduction

The World Health Organisation quoting data from April 16th, 2021, stated there were 137,866,000 cases of COVID-19 and over 2,965,000 deaths worldwide [1]. In March 2020 the UK government went into lockdown and these restrictions were relaxed from May 2020. During this time venues such as public houses, restaurants and non-essential shops were closed, and the population was mandated to stay at home wherever possible. For UK Universities this meant that virtually all student contact was restricted to online interactions.

A recent systematic review and meta-analysis has confirmed the presence of a link between disturbed sleep and psychological distress during the COVID pandemic [2]. Work conducted in China has found that the most important risk factors associated with insomnia and mental health during the COVID pandemic were being a health care worker, a woman, having an underlying illness, living in a rural area and being in contact with COVID-19 infected patients [3].

Notwithstanding the pandemic-related lockdown there are several factors which may affect dysfunctional sleep/insomnia in general, these are not exhaustive. Firstly, demographic factors- women are more likely to suffer from insomnia as are individuals who live alone, secondly, having a family history of insomnia, thirdly psychological factors such as anxiety and depression [4], fourthly, lifestyle issues such as smoking [5] and alcohol (this will be briefly expanded later), fifthly life events, such as divorce and bereavement [6] and finally physical pain [7]. There are also a series of “bad habits” which are associated with disturbed sleep, such as spending excessive amounts of time in bed, napping, medication use, sleeping during the day, alcohol, caffeine, and nicotine use. This is especially the case for shift workers [8].

Alcohol is a low-risk sedative. Johnson et al. [9] found that 15% of their US general population sample (n = 2184) used alcohol as a night-time sedative for four weeks or more. Whilst alcohol has sedative properties and may result in sleep, the quality of sleep is poor, and the individual can wake up unrefreshed. With continued use, the quality of sleep declines largely because an individual falls into a deep sleep quickly but as result there is an imbalance between slow wave and REM sleep, meaning there is a reduction in REM (deeper sleep), often the resulting in shorter sleep duration and frequent awakening. The Institute of Medicine [10] provides a summary of the architecture of typical night’s sleep.

There are several areas where the alcohol has a detrimental impact on sleep, these include sleep disordered breathing, excessive snoring, and sleep apnoea [11]. This results in a reduction in oxygen because the upper airway dilator muscles are relaxed which increases resistance in the nose and pharynx. In consequence if an individual has a sleep apnoea, they may be difficult to rouse from sleep [12]. There are other ways in which alcohol can result in disturbed sleep, it increases leg movements, (known as restless legs syndrome) [13] and the likelihood of sleep walking when combined with psychotropic medication [14]. It can also induce gastritis, oesophageal reflux, and polyuria, all of which can disturb sleep [15].

A study of Italian nurses (n = 1005) during the COVID pandemic [16] found the following prevalence statistics, sleep disturbances (71.4%), moderate anxiety (33.23%) and low self-efficacy scores (50.65%). Anxiety was positively related to poor sleep quality and low self-efficacy scores were associated with anxiety and diminished sleep quality. Females had more sleep disturbances and higher anxiety scores and lower levels of self-efficacy than males.

To date most of the research that has considered sleep, mental health and alcohol has been in veteran populations [17], students [18], and clinical populations [19]. This online survey of academic and administrative university staff and students reports sleep changes associated with the COVID lockdown and the link between demographic variables, health and wellbeing, self-efficacy, changes in alcohol consumption and daily routine. None of the participants declared sleep or other physical or mental health problems.

## 2. Methods

A small research group of interested staff who were university lecturers in health, psychology and education considered which behaviours and beliefs which they wished to investigate during the first lockdown period. By this point, they had experience of the lockdown themselves and had insights into the concerns students had over this period. These online research meetings took place online between April–May 2020.

The survey was online using Qualtrics software that was distributed to staff and students on a university sanctioned address book following ethical approval from the University of Greenwich Research Ethics Committee. It was distributed over six weeks with two-week intervals between 1 July 2020 and mid-August 2020.

### 2.1. COVID Survey

The survey collected the following demographic data: gender, age, whether an individual was a student or member of the university staff, ethnicity, caring responsibilities, and highest educational level. There were also nine items concerning routine during the lockdown period. Individuals were asked their level of agreement with the statement. These were treated as categorical variables and coded as follows: Disagree/Strongly Disagree = 1, Neither Agree or Disagree = 2, and Agree/Strongly Agree =3.

### 2.2. Sleep

This study uses the sleep score of the Nottingham Health Profile [20] as recommended by Kind and Carr-Hill [21]. Under this schema each of the five items are scored as 0 or 1 dependent upon whether the item is endorsed or not. Thus, the range of scores is 0–5 (higher scores indicating greater sleep dysfunction). This method of scoring was also used in Foster et al. [22].

### 2.3. Alcohol Consumption during Lockdown

Stein and Friedmann [15] confirmed the link between alcohol and disturbed sleep. Participants were asked if they were drinkers and then if so whether their drinking had changed during the lockdown period. Not applicable was scored 0, less than before the lockdown 1, no change 2 and more than the lockdown 3.

### 2.4. Self-Efficacy

Self-efficacy was assessed by the Generalized Self-Efficacy scale [23]. This is a widely used international instrument (designed in Germany) and its psychometric properties have been established for cross-cultural use [24]. It consists of 10 items each scored as follows, not true at all (1), hardly true (2), moderately true (3) and exactly true (4). The range of scores is 10–40 and higher scores indicate greater self-efficacy. Schwarzer [25] report a norm score of 29.48 (SD = 5.13) in 1594 US adults in which gender was evenly distributed.

### 2.5. WEMWBS

The WEMWBS (Warwick and Edinburgh Mental Health and Well-Being Scale) [26] assessed mental health and well-being. Validity and reliability have been widely tested and this measure can be used in the general population [27]. The current study uses the short-form measure which has 14 items scores none of the time (1)–all the time (5). Higher scores are indicative of better health and well-being. The range of scores is 14–70. A score of 40 or below is in a “probable depression” range, 41–44 “is possible depression”, 45–59 is “average” mental health and well-being and a score of 60 or above is indicative of “good” mental health and wellbeing [28].

## 3. Data Analysis

Initially descriptive statistics were presented and thereafter all the study variables were entered into a multinomial logit model regression entering changes in sleep scores as the dependent variables. This model was constructed using the “main-effects” command which meant that categorical and numerical variables could be combined as co-variates. The regression reports statistical probability, odds ratios and 95% confidence intervals respectively.

## 4. Results

Table 1 shows the characteristics of the study sample. Over seventy percent of the sample were women and over 80% were students. However, university staff were more likely to have caring responsibilities (n = 91, 24.7%) (n = 276, 13.9%) (Staff: Students) (*p* < 0.001). 329 (89.6%) of the carers stated they had dependent children.

### 4.1. Alcohol Consumption

Data was collected concerning alcohol consumption. 647 (36.4%) described themselves as “never drinkers” and a further (n = 272, 15.7%) answered that they typically drank less than monthly. 1774 (75.8%) replied to a question concerning whether there were any changes to their alcohol consumption during the lockdown period. 646 (36.4%) stated “not applicable”. Of those who were drinking, the highest proportion answered no change (n = 458, 25.8%), (n = 352, 19.8%) were drinking more than before the lockdown and (n = 318, 17.9%) less than the lockdown.

### 4.2. Self-Efficacy

The mean value for the self-efficacy scores (n = 1598) was 30.54 (SD = 5.20). This compares to US population adult mean norm scores (n = 1594) of 29.2 [25] so there was a high level of perceived self-efficacy. The results for each self-efficacy value are shown in Table 2. The two items that had the highest level of endorsement (highest scores) were solving problems if I try hard enough and solving problems if I invest the necessary effort.

### 4.3. Health and Well-Being

The mean health and well-being (WEMWBS) scores (n = 1571) were 45.09 (SD = 10.81), indicative of low/average health and well-being [26]. Table 3 shows the data for individual WEMWBS scores (higher scores are indicative of better health and well-being). The items were most indicative of positive health and well-being were feeling loved and being able to make your own mind about things.

### 4.4. Sleep Scores

There was a significant decline in sleep scores during the lockdown period (n = 1798) (1.68, 1.36, Mean, SD) (1.93, 1.40, Mean SD) (Prior to Lockdown) (During Lockdown) (*p* < 0.001). Table 4 shows the changes in scores for every sleep variable. Each one was (*p* < 0.001). The largest decline was in “I usually wake up in the early hours of the morning”, followed by (in descending order) “it usually takes me a long time to get to sleep” then “I usually sleep badly at night”, “I usually lie awake for most of the night” and finally “I usually take pills to help me sleep”.

### 4.5. COVID Daily Routine

The variables with the highest level of agreements with the statement were in descending order “having an area to work in away from members of the household”, “not switching on the television until a certain time “and “getting up at a regular time”. The other items, also in descending order were taking a break from the screen”, “not looking at work communications once work had stopped”, “planning the day so that there was a break from online meetings” “stopping work at 5 p.m.”, “putting on work clothes or make-up” and “ensuring a lunch break or similar was taken”.

### 4.6. Regression Findings

Table 5 shows the results of the multinomial logit model regression for all the study variables. Student has been created as a dummy variable. (Staff coded 0, Student coded 1) The non-COVID-related variables that were significantly related to changes in sleep scores in the regression were alcohol consumption, WEMWBS scores, gender, and educational level. Increased sleep scores (deterioration) were associated with lower health and well-being scores, lower educational status, and greater alcohol consumption during pandemic. Women had a greater increase in sleep scores than men. There were two COVID-related variables that were significantly related to changes in NHP sleep scores. “Not having a designated area to work in” and “Not putting on clothes and make-up” were both associated with greater sleep dysfunction during the lockdown period.

## 5. Discussion

This paper has found that there was a significant deterioration in sleep scores as assessed by the Nottingham Health Profile-sleep subscale using a scoring method suggested by Kind and Carr Hill [21] which was associated with going into extended lockdown. This was in a sample of university staff and students who completed an online survey. Women and individuals with a lower educational status were more likely to experience a deterioration in sleep scores, this was also associated increased alcohol consumption and a reduction in health and well-being scores. There were two area of COVID routine that were associated with the change in sleep scores. Both not having a designated area to work in and not putting on clothes and make-up were both associated with greater sleep dysfunction during the lockdown period. All areas of sleep that were assessed, deteriorated significantly during the lockdown period. These included indicators of sleep quality, sleep latency, sleep duration, sleep disturbance and increased use of sleep medication.

The fact that women had more disturbed sleep/insomnia associated with lockdown than men is consistent with international research [29]. However, Voderholzer et al. [30] suggest that the gender differences in insomnia who are sub-clinical can be explained by anxiety and/or depression. A summary of the research considering sleep during the lockdown period [31] suggests that stress can lead to sleep disturbance/insomnia, they point to worries about health and employment and the fact that working parents are often combining having to continue working at home with home schooling and what they termed “household errands”. These may have an impact of how many hours there are available to sleep. Other factors Altena et al. [31] point to having an impact on health and wellbeing in consequence of the lockdown include not being able to socialise with family friends or attend cultural or sporting events, lack of exercise and a tendency to eat more than usual in response to stressful situations. They also point to the importance of maintaining a routine during a lockdown period [32]. The findings of the current study suggest that the importance of having space in which to work and putting on clothes and make up are areas worth further investigation. The significant association with a deterioration of sleep scores and lower educational status is consistent with Arber et al. [33].

There was a significant deterioration in all the areas of sleep that were assessed during the lockdown period, these were sleep quality, sleep latency, sleep duration, sleep disturbance and increased use of sleep medication. In the current study increased alcohol consumption compared to drinking before the lockdown was associated with sleep dysfunction. Ebrahim et al. [34] provide a summary of the impact of alcohol in healthy volunteers on a normal night’s sleep. Even in small doses alcohol will reduce the onset of sleep latency. Over the night, alcohol results in a reduction in Rapid Eye Movement (REM) sleep and an increase in Slow Wave Sleep (SWS) which if prolonged can result in poorer sleep quality and shorter sleep duration. Work from Japan [35] in a general population sample (n = 18,205) found that 48% of men and 18% of women used alcohol as a sleep aid one or more times per week, in comparison the use of hypnotic medication was far less frequent. Using alcohol as a sleep aid was associated with “difficulty in maintaining sleep”. Alcohol is a depressant and the relationship between alcohol consumption and depression [36] and anxiety [37] is well established.

### 5.1. Impact and Possible Future Research

This paper provides insights into the impact of prolonged lockdowns of sleep, alcohol consumption and health and well-being. It suggests that these are areas that clinicians, policy makers, employers and universities and colleges should consider in the event of a prolonged lockdown or similar occurring in the future. They indicate that using the five Nottingham Health Profile Sleep scores and asking whether alcohol consumption has either reduced, remained the same or increased because of some form of traumatic event (notwithstanding prolonged lockdown) could be markers for the development of sleep and/or alcohol problems.

### 5.2. Strengths and Limitations

The main strength of this study is that it examines the link between the change in sleep scores, alcohol consumption and health and wellbeing during the COVID lockdown period in large sample of both members of university staff and undergraduate/postgraduate students. It also employs three well-established validated questionnaires, self- efficacy, sleep and health and well-being as well as other measures which require further testing to establish their psychometric properties.

However, there are limitations that need to be acknowledged. This is a large sample, but like all non-probability internet surveys it is open to questions of selectivity. The aim was to collect data concerning behaviours rather than examine specific illnesses or clinical conditions thus no questions were asked concerning illness as we felt this would be off-putting for the potential participants. The data we collected concerning alcohol consumption provides some basis for this belief. The three questions of the AUDIT C (based on alcohol consumption) [38] were included in the survey, but no participants completed all three questions they were however prepared to answer a question concerning whether there had been any changes in alcohol consumption during the lockdown period. This suggests two possibilities, firstly that the questions were seen as obtrusive and over sensitive. Or secondly that within the context of a wider internet survey (many of the sample described themselves as non or minimal drinkers), it was too complex to complete. There are other reasons why these findings should be treated with caution. Most of the sample were non-drinkers and furthermore the reported changes in alcohol consumption were not quantified and based on self-report. The final limitation is that the survey is based on self-report of sleep status so that no PSG measures were used.

### 5.3. Recommendation for Practice as a Result of the Study

Although the COVID lockdown occurred in 2020 this paper provides insights into the importance of maintaining healthy sleep patterns during extended lockdown periods especially for college students. Women are more likely to be adversely affected and there should be some education concerning how to maintain positive health and well-being with an emphasis on the possible impact of alcohol upon this. It should also be noted that whilst prolonged lockdowns are unlikely, hybrid working; that is a mix of working from home and going into the office is likely to become the norm and further research is required concerning the impacts of this societal shift. In particular the role played by maintaining a routine in enhancing health and wellbeing.

## 6. Conclusions

This study confirms the link between sleep, alcohol consumption, (despite the fact a significant proportion of the sample were non or minimal drinkers), maintaining a healthy routine and health and well-being in a group of university staff and students. It provides pointers for universities, colleges and employers in general concerning how individuals experience prolonged lockdowns and a degree of isolation and how they could support them.

## Figures and Tables

**Table 1:** Description of the study sample (n = 2341).

Variable	Number	Percentage
**Gender**		
Male	631	26.9
Female	1710	73.1
**Classification**		
Students	1972	84.2
University Staff	369	15.8
**Student Status (n = 1962)**		
First Year	779	39.7
Second Year	409	20.8
Third Year	359	18.3
Masters Level	364	18.6
Doctorate	51	2.8
**Highest Educational Status**		
GCSE only or no qualifications	583	24.9
BTEC/Access/Further Education or equivalent	977	41.8
A Level or equivalent	152	6.5
University or College Degree	362	15.5
MSc/MA Degree	15	<1
Doctorate	251	10.7
**Ethnicity**		
White	2114	90.5
Asian	137	5.9
Mixed	85	3.6
**Carers**	367	15.7
	**Mean**	**SD**
Age	29.3	12.9
NHP Sleep prior to Lockdown (n = 1808)	1.7	1.4
NHP Sleep during lockdown (n = 1798)	1.9	1.4
Self-Efficacy (n = 1598)	30.5	5.2
WEMWBS (Health and Well-Being) (n = 1571)	45.1	10.8

**Table 2:** Self-Efficacy Scores for each item.

Variable	Mean	SD
I can always manage to solve difficult problems if I try hard enough (n = 1631)	3.31	0.64
If someone opposes me, I can find means and ways to get what I want (n = 1627)	2.68	0.76
It is easy for me to stick to my aims and accomplish my goals (n = 1624)	3.00	0.72
I am confident that I could deal efficiently with unexpected events (n = 1629)	3.04	0.75
Thanks to my resourcefulness, I know how to handle unforeseen circumstances (n = 1629)	3.00	0.72
I can solve most problems if I invest the necessary effort (n = 1628)	3.31	0.66
I can remain calm when facing difficulties because I can rely on my coping abilities (n = 1624)	3.00	0.84
When I am confronted with a problem, I can usually find several solutions (n = 1627)	3.04	0.71
If I am in trouble, I can usually think of a solution (n = 1626)	3.11	0.69
I can usually handle whatever comes my way (n = 1626)	3.07	0.73

**Table 3:** WEMWBS Scores for each item (n = 1571).

Variable	Mean	SD
I’ve been feeling optimistic about the future	3.11	1.01
I’ve been feeling useful	3.14	1.07
I’ve been feeling relaxed	2.98	1.04
I’ve been feeling interested in other people	3.19	1.07
I’ve had energy to spare	2.96	1.11
I’ve been dealing with problems well	3.34	0.97
I’ve been thinking clearly	3.34	1.00
I’ve been feeling good about myself	3.10	1.11
I’ve been feeling close to other people	3.11	1.08
I’ve been feeling confident	3.13	1.07
I’ve been able to make my own mind about things	3.56	0.99
I’ve been feeling loved	3.57	1.12
I’ve been interested in new things	3.37	1.08
I’ve been feeling cheerful	3.18	1.03

**Table 4:** Changes in NHP Sleep Scores.

Sleep Item	Prior to Lockdown (n = 1808)		During Lockdown (n = 1799)		*p*
	Mean	SD	Mean	SD	
I usually take pills to help me sleep	0.06	0.23	0.08	0.26	<0.001
I usually wake up in the early hours of the morning	0.50	0.50	0.53	0.50	<0.001
I usually lie awake for most of the night	0.30	0.45	0.38	0.48	<0.001
It usually takes me a long time to get sleep	0.46	0.50	0.54	0.50	<0.001
I usually sleep badly at night	0.35	0.47	0.43	0.49	<0.001

All analyses were Two-Tailed Wilcoxon Z Test.

**Table 5:** Results from a multinomial logit model regression for Study Variables entering change in NHP Sleep Scores as the dependent variable.

	B	Standard Error	t	95% Confidence Intervals	*p*
Age	0.000	0.004	(−)0.104	(−)0.007–0.007	0.917
Gender	0.177	0.071	2.503	0.038–0.316	0.012
Student	0.234	0.124	1.889	(−)0.009–0.478	0.059
Educational Level	0.080	0.029	2.767	0.023–0.137	0.006
Ethnicity	0.057	0.061	0.928	(−)0.063–0.176	0.353
Carer	0.124	0.107	1.160	(−)0.086–0.335	0.246
Alcohol Consumption	0.117	0.032	3.648	0.054–0.181	<0.001
Self-Efficacy	0.014	0.008	1.715	(−)0.002–0.030	0.087
WEMWBS	(−)0.020	0.005	(−)4.525	(−)0.029–(−)0.012	<0.001
**COVID Routine Items**					
I have/had an area where I can work away from other members of the household	(−)0.129	0.037	(−)3.491	(−)0.201–(−)0.056	<0.001
I get/got up at my regular time	0.025	0.044	0.533	(−)0.062–0.111	0.580
I put on my work clothes/makeup	0.184	0.071	2.596	0.045–0.323	0.010
I take/took regular breaks from a screen	(−)0.066	0.047	(−)1.409	(−)0.159–0.026	0.159
I plan/planned my day so that I can be away from online meetings	(−)0.036	0.049	(−)0.726	(−)0.132–0.061	0.468
I ensure/ensured I have a lunch break or similar	(−)0.038	0.041	(−)0.927	(−)0.119–0.043	0.354
I stop/stopped work at 5 p.m.	(−)0.033	0.042	(−)0.798	(−)0.115–0.049	0.425
When I stop/stopped work I do not look work communications	0.058	0.040	1.439	(−)0.021–0.137	0.151
I do/did not switch on the television or similar until a certain time.	0.069	0.041	1.674	(−)0.012–0.150	0.094

B = Odds Ratios.

## Data Availability

The data set is available from the corresponding author on request.
